# Preintubation Machine-Delivered Pressure Support Ventilation With Positive End-Expiratory Pressure Versus Manual Bag-Mask Ventilation for Oxygenation in Overweight and Obese Patients: A Randomized, Pilot Study

**DOI:** 10.7759/cureus.45185

**Published:** 2023-09-13

**Authors:** Pragadeshwaran Rajendran, Habib Md R Karim, Chinmaya K Panda, Praveen K Neema, Samarjit Dey

**Affiliations:** 1 Liver Transplant Anaesthesia and Critical Care, Gleneagles Global Health City, Chennai, IND; 2 Anesthesiology, Critical Care, and Pain Medicine, All India Institute of Medical Sciences, Deoghar, Deoghar, IND; 3 Anaesthesiology, Critical Care, and Pain Medicine, All India Institute of Medical Sciences, Raipur, Raipur, IND; 4 Cardiac Anaesthesiology, Amrita Institute of Medical Sciences and Research Centre, Kochi, IND; 5 Anaesthesiology, All India Institute of Medical Sciences, Mangalagiri, Mangalagiri, IND

**Keywords:** overweight, obese, body weight, tracheal intubation, oxygen, airway resistance, tidal volume, positive pressure respiration

## Abstract

Background: Noninvasive positive pressure ventilation (NIPPV) maintains mean airway pressures well, and its usability for preoxygenation is well described. Anesthesia machine-delivered NIPPV-based preoxygenation has recently been evaluated against the traditional manual bag-mask ventilation (BMV). The efficiency of such a technique over the traditional one is yet to be established well. The present study evaluated the feasibility of machine-delivered preoxygenation using pressure support ventilation (PSV) with positive end-expiratory pressure (PEEP) and compared the effectiveness with BMV.

Methods: Thirty overweight and obese adults belonging to the American Society of Anesthesiologist's physical status I-II were randomized to receive PSV+PEEP or BMV for preintubation preoxygenation targeted to a fraction of expired oxygen (FeO_2_)_ _of 85% and 90% or for a maximum period of five minutes, whichever came first. Postintubation, the patient was observed for the time taken until 1% desaturation without ventilation. Arterial blood gases, respiratory variables, FeO_2 _achieved, and different times were collected and compared.

Results: The baseline characteristics and arterial blood gases were similar between the two groups. The PSV+PEEP group had consistent and favorable tidal volume and airway pressure delivery. The difference in time to reach a FeO_2_ of 85% between the two groups was not statistically different. Only two patients achieved a FeO_2_ of 90% in the PSV+PEEP group versus none in the BMV group. However, partial pressure of oxygen at 1% desaturation (217.42±109.47 versus 138.073±71.319 mmHg, p 0.0259) was higher in the PSV+PEEP group. Similarly, the time until 1% desaturation was significantly prolonged in the PSV+PEEP group (206.6±76.952 versus 140.466±54.245 seconds, p 0.0111).

Conclusion: The present pilot study findings indicate that preintubation machine-delivered PSV+PEEP-based preoxygenation is feasible and might be more effective than traditional BMV in overweight and obese patients.

## Introduction

Preoxygenation before general anesthesia (GA) induction and endotracheal intubation (ETI) increases oxygen stores in the lungs' functional residual capacity (FRC) and various other body tissues [1]. It prolongs the apnea time - from the cessation of breathing or ventilation until the onset of significant arterial desaturation [2]. The risk of critical desaturation increases exponentially in case of unanticipated difficult intubation, where preoxygenation's importance is paramount. Overweight and obese patients gain a prominent position as the difficult intubation in obese patients is nearly doubled compared to the nonobese population [3]. Furthermore, physiological changes in overweight and obese patients like reduced lung volumes and capacities, closing capacity nearing or even falling within tidal breathing, particularly in the supine position, and decreased compliance put such patients at risk of rapid desaturation and hypoxia events every day during intubation [4]. With maximal preoxygenation, the time to oxyhemoglobin desaturation below 80% can vary from nine minutes in a healthy, nonobese adult to three minutes or less in children or obese adults [5].

Over time, several techniques have been proposed and studied to enhance preoxygenation in obese patients. Noninvasive positive pressure ventilation (NIPPV), combining the benefits of pressure support ventilation (PSV) and positive-end expiratory pressure (PEEP), keeps the lungs open during the entire respiratory cycle above the assistance of ventilation. Fogarty et al. conducted a study to determine the efficacy of feedback-controlled mask NIPPV over traditional bag-mask ventilation (BMV) and showed the feasibility of its preoxygenation use [6]. However, the clinical applicability - whether the increased delivered volume observed also improved or optimized oxygenation- must be evaluated [7]. The present pilot study was designed to evaluate the effectiveness and feasibility of machine-delivered preoxygenation in overweight and obese patients and compare the efficiency of machine-delivered PSV and PEEP with traditional BMV preoxygenation.

## Materials and methods

Settings and design

The present randomized, parallel-group, single-blind, clinical pilot study was conducted in a tertiary care institute in India. The postgraduate thesis review committee of the institute reviewed the study. Furthermore, the study was evaluated by the Institute (All India Institute of Medical Sciences, Raipur) Ethical Committee and approved (No. 1051/IEC-AIIMSRPR/2020 dated 11.05.2020). The study was conducted according to the Declaration of Helsinki, and Good Clinical Practice was followed. Trail Registry was done prospectively (CTRI/2020/06/026203) at Clinical Trail Registry - India, and all participants were enrolled following informed written consent.

Participants

Participants aged 18 to 65 with a body mass index (BMI) of 25 - 39.99 kg/m^2 ^and room air peripheral oxyhemoglobin saturation (SpO_2_) of more than 95% undergoing elective surgeries under GA with ETI were enrolled in the study. Exclusion criteria were patient's refusal, active acute or chronic respiratory conditions, respiratory failure, airway obstruction, heart failure, patients with anticipated difficult intubation (having two feature positive of the more prominent incisor (bucked teeth), inter-incisor distance < 3 cm, and thyromental distance < 5.5 cm), inability to get a proper fit mask, hypothyroidism, hyperthyroidism, and pregnancy.

Randomization, allocation, and blinding

Participants were randomly allocated to either of the two groups (Group A- PSV with PEEP of 5 cm of water versus Group B- Manual BMV). A simple randomization technique was followed. Sequence generation was done using the online epidemiological tool www.epitools.com by the guide of the project. Allocation was based on the selection of the number by the patient, and concealment was maintained by keeping the random sequence centralized with the guide. A total of 44 random numbers were generated at the beginning of the study and kept centrally with the study guide. While participants were kept blind to the procedure and group allocation, the same was not feasible for the investigators due to the inherent nature of the interventions. Although the statistician who performed the data analysis was blind to the intervention, the master chart and statistical review were also conducted by the study guide, who was not blind to the intervention. 

Intervention technique

In the operating room, patients were positioned with their head end elevated and necks extended. The American Society of Anesthesiologists (ASA) standard monitoring was initiated, and pulse-oximetry of the same make was used in all patients. GA induction was standardized. Fresh gas flow was set as minute ventilation + 500 ml. Patients were induced using propofol (2-2.5 mg/Kg) and nalbuphine (0.15 mg/Kg). Vecuronium 0.1 mg/Kg was administered only after ensuring ventilation. Group allocation was disclosed to the intraoperative anesthesia team at this point. In the group with manual BMV, patients were ventilated by a person with a minimum of six months of training in anesthesia, and ventilation was at the performer's discretion. In the PSV+PEEP group, the PSV with a backup rate equal to 8ml/Kg IBW x 14 breaths per minute was given using the Dräger (Drägerwerk AG & Co., Lübeck, Germany) Primus anesthesia machine and circuit. Pressure support was adjusted as required to achieve the volume of approximately 8mL/kg ideal body weight (IBW). A PEEP of 5 cm water was added; 100% oxygen was used throughout. Both the groups received ventilation using an appropriate size tight-fitting mask. After five minutes, 0.5mg/kg propofol was administered as a bolus, and the patient was intubated. The correct placement of the ET tube was confirmed by capnography and five-point auscultation (five breaths). After confirmation, the ET tube was left open to the atmosphere and observed until 1% desaturation. At this point of desaturation by 1% or at five minutes, whichever is earlier, one arterial blood gas (ABG) sample was taken by a well-experienced (having more than two years of experience) person for analysis. It ended the intervention, and after that, standard management was followed for both ventilation and peri-operative management as per institutional protocol for both groups.

Outcome variables

Preoperative patient demographics, SpO_2_, hemodynamics, and hemoglobin were noted. A baseline ABG was obtained before induction for all recruited participants. Tidal volume, minute ventilation, SpO_2_, fraction of inspired oxygen (FiO_2_), fraction of expired oxygen (FeO_2_), end-tidal carbon-di-oxide (EtCO_2_), peak airway pressure, and alarms were noted each minute until five minutes during the intervention phase in both groups. Also, this phase noted the time taken for pre-oxygenation till FeO_2_ of 85% and 90%. Furthermore, time taken for 1% desaturation was noted, followed by an ABG, as mentioned earlier.

Sample size calculation

The sample size for the present pilot study was calculated using http://www.crutzen.net/n.htm based on the formula by Viechtbauer et al. [8]. We hypothesized a 20% probability of detecting the difference and calculated the sample with 95% confidence. Considering the possible 10% drop-out or exclusion and a design effect of 1.0 (considering randomized design), the sample size was 15 per group. However, we planned a per-protocol analysis, and therefore, it was decided to randomize a maximum of 40 patients to recruit at least 15 participants in each arm, whichever occurs first. 

Data management and statistics

The master chart was prepared in Microsoft Excel (Microsoft Corporation, Washington, United States). Categorical variables were presented on a number and percentage scale. Continuous data were presented as mean ± standard deviation (SD). Group comparison was performed using unpaired student t-tests, Fisher’s exact test, and another appropriate test if the data distribution is other than normal. A p-value less than 0.05 was considered statistically significant. Instat software (GraphPad Prism, La Jolla, California, United States) was used for statistics.

## Results

**Figure 1:**
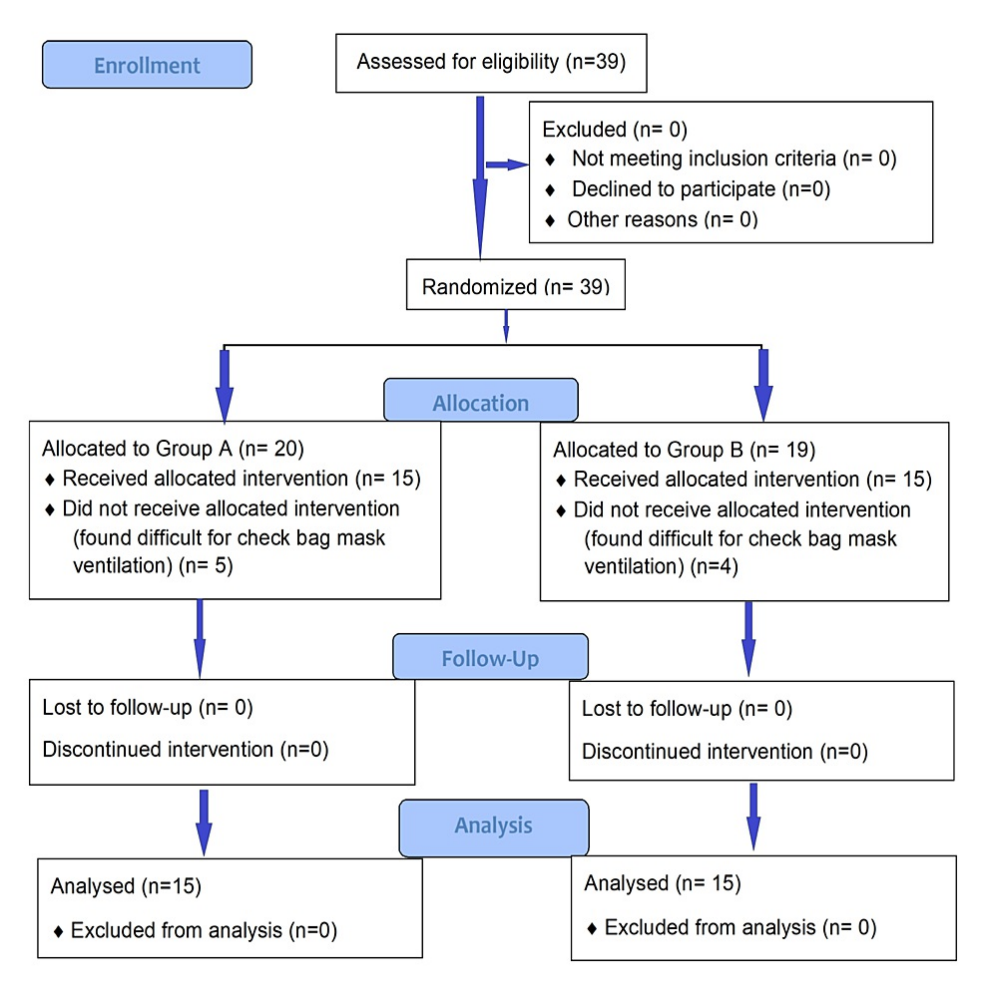
Consolidated Standards of Reporting a Trial (CONSORT) 2010 flow diagram of participants.

Between January 2021 and November 2021, thirty-nine patients were screened, 20 in group A and 19 in group B, and were found suitable and enrolled in this study. Five patients from group A and four from group B were excluded due to difficult bag-mask ventilation. The study did not require any follow-up, and data were complete for all cases. The remaining 30 (15 in each group) were analyzed (Figure 1).

The baseline characteristics of the two groups were similar in terms of age, gender, height, weight, BMI, IBW, heart rate, respiratory rate, SpO_2_, preoperative hemoglobin concentration, National Institute Health and Care Excellence (NICE) grading, and ASA-physical status classification (Table 1). Arterial blood gases also did not differ between the two groups.

**Table 1:** Characteristics of the participants before preoxygenation presented in either number (percentage) or mean ± SD scale; p-value <0.05 is significant. N: Total number, PaCO_2_: partial pressure of arterial carbon dioxide, PaO_2_: partial pressure of arterial oxygen, SpO_2_: peripheral oxyhemoglobin saturation, SD: standard deviation.

Parameters	Group A [N=15]	Group B [N=15]	p-value
Age (years)	48.93 ± 11.91	51.33 ± 7.48	0.5142
Gender (Male/Female)	9 (60.0) / 6 (40.0)	8 (53.33) / 7 (46.67)	>0.99
Height (cm)	160.73 ± 7.58	160.6 ± 8.87	0.8266
Weight (kg)	75.63 ± 9.15	75.6 ± 7.71	0.9915
Body mass index (kg/m^2^)	29.24 ± 2.71	29.64 ± 3.7	0.7341
Ideal body weight (kg)	56.13 ± 7.51	55.66 ± 8.36	0.8734
Haemoglobin (g/dl)	13.14 ± 1.35	12.17 ± 1.74	0.1017
Heart rate (per min)	84.266±8.722	88.266±15.568	0.3927
Respiratory rate (per min)	15.2±1.699	16.466±2.326	0.0966
SpO_2_	98.2±1.781	97.8±1.568	0.5191
Diabetes n (%)	5 (33)	3(20)	0.6817
Hypertension	2 (13)	2(13)	1.0000
Obesity	4 (27)	2(13)	0.6513
Obstructive Sleep Apnea	1 (7)	-	1.0000
PaO_2_ (mmHg)	87.397 ± 8.576	89.14 ± 6.181	0.5284
PaCO_2_ (mmHg)	33.146 ± 4.505	33.785 ± 5.086	0.7185
pH	7.44 ± 0.0384	7.429 ± 0.052	0.5074

The differences in minute ventilation and peak pressure between the two groups at various time intervals were statistically indifferent. However, there were significantly minimal variations from ideal minute ventilation with Group A. The RR and RR variations in percentage scale were significantly different between the two groups at various time intervals. The tidal volume and variations were also significantly different between the two groups at various time intervals (Table 2). The PSV+PEEP group showed favorably.

**Table 2:** Ventilatory characteristics and parameters during preoxygenation presented in mean ± SD scale; p-value <0.05 is significant. N: Total number, NA: not applicable, SD: standard deviation.

Parameters	Group A [N=15]	Group B [N=15]	p-value
Respiratory rate - 1 min	13.93±0.4577	19.066±6.713	0.0063
Respiratory rate - 2 min	14.066±0.258	17.466±4.853	0.0114
Respiratory rate - 3 min	14	18.26±4.743	NA
Respiratory rate - 4 min	14	17.13±4.121	NA
Respiratory rate - 5 min	14	18.26±3.615	NA
Respiratory Rate Var Max	0.476±1.844	32.896±37.267	0.0022
Respiratory Rate Var Min	7.857±9.101	22.469±13.466	0.0017
Tidal volume - 1 min	436.93±92.710	322.66±112.32	0.0051
Tidal volume - 2 min	461.6±96.524	326.93±112.57	0.0015
Tidal volume - 3 min	446.266±82.619	303.533±82.019	<0.0001
Tidal volume - 4 min	431.93±78.6	307.6±74.943	0.0001
Tidal volume - 5 min	437.06±91.622	318.4±90.938	0.0013
Tidal volume - Var Max	16.697±9.641	20.872±17.099	0.4170
Tidal volume - Var Min	9.764±11.065	40.292±17.683	<0.0001

The difference in time taken to reach a FeO_2 _of 85% between the two groups was not statistically significant. Only two patients achieved a FeO_2_ of 90% in Group A. No patient achieved the target FeO_2_ of 90% in Group B. The time until 1% desaturation was significantly prolonged in Group A. The difference in pH at the time of 1% desaturation was not statistically significant between the two groups. However, the two groups had statistically significant differences in PaO_2_ and PaCO_2 _at 1% desaturation (Table 3).

**Table 3:** Comparison of oxygenation, ventilation, and arterial blood gases at 1% desaturation presented in mean ± SD scale; P-value <0.05 is significant. N: Total number, PaCO_2_: partial pressure of arterial carbon-di-oxide, PaO_2_: partial pressure of arterial oxygen, SD: standard deviation.

Parameters	Group A [N=15]	Group B [N=15]	p-value
PaO_2_ (mmHg)	217.42±109.47	138.073±71.319	0.0259
PaCO_2_ (mmHg)	41.766±8.032	51.244±9.293	0.0058
pH	7.3479±0.0377	7.3474±0.0378	0.9733
Time to 85% FeO_2_ (seconds)	189.166±56.69	207.142±42.146	0.3637
Time to 1% desaturation (seconds)	206.6±76.952	140.466±54.245	0.0111

**Figure 2:**
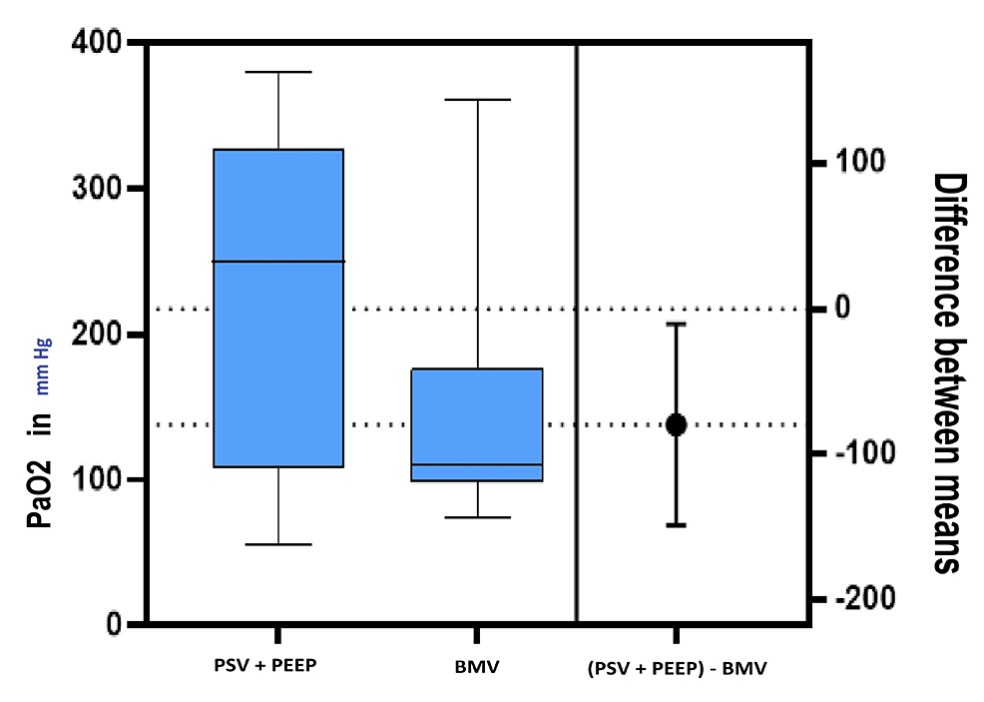
The mean, dispersions, and difference between the means for the PaO2 at 1% desaturation. BMV: Bag mask ventilation,  PaO_2_: partial pressure of arterial oxygen, PSV: pressure support ventilation, PEEP: positive end-expiratory pressure.

**Figure 3:**
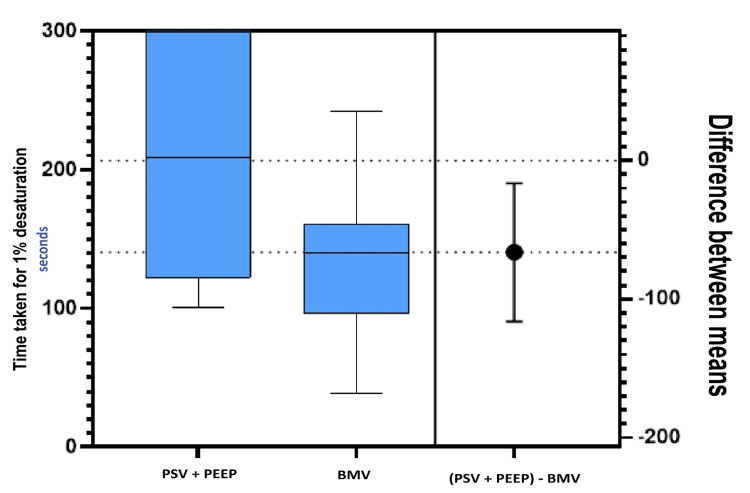
The mean, dispersions, and difference between the means for the apnea time until 1% desaturation. BMV: Bag mask ventilation, PSV: pressure support ventilation, PEEP: positive end-expiratory pressure.

The differences in the means for PaO_2_ at 1% desaturation (Figure 2) and time taken until 1% desaturation during apnea are presented in Figure 3.

## Discussion

The present pilot study did not find notable differences in all aspects examined, but a few results have noteworthy practical implications and clinical applications. The first is the significant variation of tidal volumes and RR in the traditional BMV group and the optimal and consistent delivery of these, including airway pressures in the PSV+PEEP group. The second critical finding was the significantly increased PaO_2_ even at the onset of desaturation in the PSV+PEEP group. Thirdly, the time taken for 1% desaturation was significantly higher during apnea in the PSV+PEEP group compared to the traditional BMV group. The experimental model by Hardman et al. found that hypoventilation significantly shortened the desaturation time [9]. Our clinical finding corroborates with it; although MV was similar, a lower tidal volume and higher RR in the traditional BMV led to lower alveolar ventilation in our study.

NIPPV improves preoxygenation efficiency by increasing mean airway pressures (Pmean-aw). Pmean-aw is directly related to tidal volume, RR, inspiratory time, Ppeak, and PEEP. A consistently increased Pmean-aw could be achieved with consistent tidal volume, RR, and a constant PEEP delivered through the machine for preoxygenation. Delay et al. demonstrated a faster time to achieve a FeO_2_ of 90% in morbidly obese patients by increasing Pmean-aw [10]. In our study, the PSV+PEEP group had optimal and consistent Pmean-aw pressures, contributing to better timing to achieve preoxygenation.

End-tidal alveolar oxygen as measured as FeO_2_ and arterial partial pressure of oxygen (PaO_2_) have been used as surrogates to evaluate the preoxygenation effectiveness [11,12]. Although our study was statistically insignificant, the time to reach a FeO_2_ of 85% was shorter in the PSV+PEEP group. Furthermore, the only two patients who reached target FeO_2_ of 90%, considered an objective target for preoxygenation [1], were in the PSV+PEEP group. The drop in saturation during apnea is another surrogate to compare the preoxygenation efficiency [13]. Even when target FeO_2_ was not achieved, probably because of the administration of an inhalational agent during this phase in our cohort, PaO_2_ was significantly higher in the PSV+PEEP group at the onset of desaturation. This finding indicates that PSV+PEEP might extend the margin of safety during apnea time before endotracheal intubation.

The other notable finding of the present study is that the peak inspiratory pressures (Ppeak) did not exceed 14 cmH_2_O in any patient in the study group, and the inspiratory flow was fixed. Studies on gastric insufflation related to Ppeak have detected gastric insufflation at 20 cm of water by auscultation and at 15 cm of water by ultrasonography [14,15]. Therefore, PSV+PEEP might confer additional advantages of negating pulmonary aspiration risk and preventing airway compromise resulting from gastric insufflation [16].

Furthermore, NIPPV-based preoxygenation has some favorable technicalities. An important factor contributing to preoxygenation efficacy reduction is the reduction in FiO_2_ due to leaks [17]. NIPPV-based preoxygenation has the potential to free one working hand that would be vital in an emergency, human resource-limited setting and when a two-handed technique for providing an optimal mask seal and airway maneuvering in an anticipated or unanticipated difficult airway is required.

Although the impact of a few minutes of such ventilation on clinical outcomes is likely negligible, a favorable mechanism is always desirable. Nevertheless, an ideal ventilatory strategy aims to prevent barotrauma, limit regional hyperinflation, ensure adequate gas exchange, and recruit collapsed alveoli without injuring normal compliant lung units [18]. NIPPV would be ideal in overweight and obese patients because manual ventilation is often unpredictable and relatively unconstrained, even in experienced hands.

The study had some limitations as well. Although we claim that preoxygenation is machine-delivered, PSV required for different patients differed at different points. Initial adjustment of pressure support was required to achieve tidal volume according to the patient's IBW. Nevertheless, this limitation can be overthrown easily using information technology; closed-loop technology to automatically adjust the PSV is a practical solution. Secondly, even if the time to desaturation was significantly prolonged, the increase in FRC with PS+PEEP was not sustained after removing the mask for laryngoscopy. It calls for prospects to design a device or technique to sustain the increased FRC.

## Conclusions

Based on the findings of our pilot study, we conclude that a preintubation machine-delivered NIPPV-based preoxygenation using PSV+PEEP is a safe, effective, and feasible option. Furthermore, it might be better than manual BMV in overweight and obese patients. However, large, multi-center research will be needed to confirm the same. Our study limitations and technicality indicate a need to find a measure to sustain the increased FRC during apnea achieved by PEEP. Software development to provide feedback-controlled pressure adjustments might be helpful.

## References

[1] Nimmagadda U, Salem MR, Crystal GJ (2017). Preoxygenation: physiologic basis, benefits, and potential risks. Anesth Analg.

[2] Herriger A, Frascarolo P, Spahn DR, Magnusson L (2004). The effect of positive airway pressure during pre-oxygenation and induction of anaesthesia upon duration of non-hypoxic apnoea. Anaesthesia.

[3] Shailaja S, Nichelle SM, Shetty AK, Hegde BR (2014). Comparing ease of intubation in obese and lean patients using intubation difficulty scale. Anesth Essays Res.

[4] Shaw M, Waiting J, Barraclough L, Ting K, Jeans J, Black B (2021). Airway events in obese vs. non-obese elective surgical patients: a cross-sectional observational study. Anaesthesia.

[5] Dixon BJ, Dixon JB, Carden JR (2005). Preoxygenation is more effective in the 25 degrees head-up position than in the supine position in severely obese patients: a randomized controlled study. Anesthesiology.

[6] Fogarty M, Kuck K, Orr J, Sakata D (2020). A comparison of controlled ventilation with a noninvasive ventilator versus traditional mask ventilation. J Clin Monit Comput.

[7] Karim HM, Obradović D, Esquinas AM (2020). Preintubation feedback controlled machine delivered noninvasive ventilation versus human delivered traditional mask ventilation: is human performance inferior to machine?. J Clin Monit Comput.

[8] Viechtbauer W, Smits L, Kotz D, Budé L, Spigt M, Serroyen J, Crutzen R (2015). A simple formula for the calculation of sample size in pilot studies. J Clin Epidemiol.

[9] Hardman JG, Wills JS, Aitkenhead AR (2000). Factors determining the onset and course of hypoxemia during apnea: an investigation using physiological modelling. Anesth Analg.

[10] Delay JM, Sebbane M, Jung B (2008). The effectiveness of noninvasive positive pressure ventilation to enhance preoxygenation in morbidly obese patients: a randomized controlled study. Anesth Analg.

[11] Bhatia PK, Bhandari SC, Tulsiani KL, Kumar Y (1997). End-tidal oxygraphy and safe duration of apnoea in young adults and elderly patients. Anaesthesia.

[12] Drummond GB, Park GR (1984). Arterial oxygen saturation before intubation of the trachea. An assessment of oxygenation techniques. Br J Anaesth.

[13] Baraka AS, Taha SK, Aouad MT, El-Khatib MF, Kawkabani NI (1999). Preoxygenation: comparison of maximal breathing and tidal volume breathing techniques. Anesthesiology.

[14] Brimacomb J, Keller C, Kurian S, Myles J (2002). Reliability of epigastric auscultation to detect gastric insufflation. Br J Anaesth.

[15] Bouvet L, Albert ML, Augris C (2014). Real-time detection of gastric insufflation related to facemask pressure-controlled ventilation using ultrasonography of the antrum and epigastric auscultation in nonparalyzed patients: a prospective, randomized, double-blind study. Anesthesiology.

[16] Wenzel V, Idris AH, Dörges V (2001). The respiratory system during resuscitation: a review of the history, risk of infection during assisted ventilation, respiratory mechanics, and ventilation strategies for patients with an unprotected airway. Resuscitation.

[17] McGowan P, Skinner A (1995). Preoxygenation--the importance of a good face mask seal. Br J Anaesth.

[18] Marcy TW (1993). Barotrauma: detection, recognition, and management. Chest.

